# Research progress in neuropathic pain after spinal cord injury: a bibliometric study from 2013 to 2024

**DOI:** 10.3389/fneur.2024.1486584

**Published:** 2024-12-24

**Authors:** Qin Huiqing, Lin Weishan, Gui Yuchang, Tang Yun, Xu Jianwen

**Affiliations:** Department of Rehabilitation Medicine, The First Affiliated Hospital of Guangxi Medical University, Nanning, Guangxi, China

**Keywords:** neuropathic pain, research progress, bibliometrics, spinal cord injury, CiteSpace

## Abstract

**Background:**

The incidence of neuropathic pain (NP) after spinal cord injury (SCI) is quite high. This pain is clinically challenging to treat and has an debilitating effect on patients. In recent years, NP is a popular topic of research and a number of relevant articles have been published in academic journals. The purpose of this article is to analyze the global research trend of NP after SCI using bibliometric methods.

**Methods:**

The literature was screened from 2013 to 2024 based on the Web of Science core collection (WOSCC). These publications, including annual publications, journals, authors, references, and keywords via CiteSpace, were analyzed in order to help understand the current research direction and hotspots in this field.

**Results:**

A total of 2022 publications were included in the analysis. The results showed that an overall upward trend in the number of publications in the study period. The top five productive journals are *Spinal Cord, Journal of Neurotrauma, Pain, Experimental Neurology*, and *Journal of Spinal Cord Medicine*, the journals related to spinal cord or pain. The top five most productive scholars are Armin Curt, Michael G. Fehlings, Wu Junfang, John L. K. Kramer, and Farinaz Nasirinezhad. Keyword bursts showed that *signaling pathway, neuroinflammation, neuralgia, spinal cord stimulation, inhibition, and depression* have become new research hotspots in the field of NP after SCI.

**Conclusion:**

This study provides a basis for the study of pain after SCI. It summarizes past research on NP following SCI and offers valuable reference data for further exploration of research trends and issues of focus in this field.

## 1 Introduction

Spinal cord injury (SCI) is a common central nervous system disease, which results in motor dysfunction below the injury level and other common complications such as muscle spasm, pain, and bladder dysfunction that impair the quality of life of patients (1). Pain, especially neuropathic pain (NP), is considered to be the most difficult pain after spinal cord injury and a powerful predictor of quality of life decline after SCI. Because it is intractable with conventional therapy, it poses difficulty in clinical treatment and needs to be solved urgently (2). Approximately 53% of patients with spinal cord injury develop neuropathic pain, which is more common below the level of lesion in patients with tetraplegia, older patients, and at 1 year post-injury (3). NP persists over an extended duration and poses formidable challenges in terms of treatment. Its enduring nature significantly impedes the rehabilitation progress of patients, exerting a detrimental influence on mood, sleep, overall quality of life, social engagements, recreational pursuits, and occupational pursuits. Moreover, it frequently imposes a substantial economic burden on the healthcare system, families, and society at large (4, 5).

NP, usually described as a burning or shooting with unusual tingling, crawling, or electrical sensations (dysesthesiae) (6), represents a prevalent chronic condition observed in clinical settings. Currently, the pathogenesis of NP remains inadequately understood, and there is a deficiency of effective targeted treatments. To alleviate the deficiency in quantitative analysis of NP following SCI research, the objective of this study is to establish a foundation for comprehensive scientific exploration of post-SCI pain spanning the last 12 years (2013–2024). By comprehensively examining the advances in studies of NP observed in the context of SCI during this time frame, this article aims to identify focal points of the current research, thereby providing a theoretical groundwork for subsequent investigations. In the medical domain, there is a burgeoning trend in the utilization of CiteSpace for scholarly articles, with a notable increase in studies focused on the trajectory of pain (7, 8).

Bibliometrics is a quantitative statistical analysis tool employed to scrutinize and comprehend research trends, playing a pivotal role in both theoretical and practical information science research (9, 10). Utilizing bibliometric methods allows for the swift elucidation of literature characteristics, analysis, and a comprehensive understanding of the development processes and research focal points within specific fields. CiteSpace stands as a frequently utilized software application for bibliometric analysis. In this study, we conducted a bibliometric analysis of publications on NP after SCI via CiteSpace 5.7 R5. The prevailing global research trend on NP after SCI encompasses various facets: publications, journals, authors, references, and keywords.

## 2 Methods

### 2.1 Search strategy

The publications considered in this investigation spanned the past 12 years (2013–2024) and were retrieved from the Science Citation Index Expanded (SCI-Expanded) within the Web of Science (WoS). The search strategy employed was as follows: [(TS = (“spinal cord injury” OR “spinal cord injuries” OR “spinal cord trauma” OR “SCI”)) AND TS = (“Neuralgia” OR “neuropathic pain “OR “allodynia” OR “neuralgic pain” OR “nervous pain” OR “nerve pain” OR “hyperalgesia”)].

### 2.2 Inclusion criteria and exclusion criteria

According to the inclusion and exclusion criteria established by Chenchaomei (11), publications related to NP after SCI, including both articles and reviews published in various academic journals, were incorporated. A total of 2024 articles were identified from the Web of Science Core Collection (WOSCC). Exclusions comprised early access, proceeding articles, book chapters, retracted publications, and news items. After excluding two articles, 2022 articles remained. No additional specific limitations were imposed, except that the chosen language was English.

### 2.3 Analysis tool

CiteSpace is a bibliometric analysis Java-based visualization software crafted by Professor Chaomei Chen (12). The software version employed in this research is CiteSpace 5.7 R5, a version subject to continuous updates. The parameters configured for CiteSpace in this study were as follows: a time-slicing approach covering January 2013 to December 2024 (1 year per slice), all options in the term source selected, one node type chosen at a time, selection criteria (top 30 or 50 objects), and pruning using Pathfinder. Nodes and links were utilized to generate visualization knowledge maps. Each node on the map denoted an element under analysis, such as a cited journal, country, or author. The node’s size reflected the frequency of citation, with different-colored nodes representing different years. Connection lines between nodes indicated co-occurrence or co-citation relationships, with line thickness signifying the strength of the relationship and color corresponding to the first co-occurrence or co-citation time of nodes. Colors ranging from cool to warm represented early to recent occurrences. Centrality, also known as betweenness centrality, was employed, considering nodes with high centrality (>0.1) as potential turning or pivotal points in the field. When the default number of network nodes in CiteSpace exceeded 350, the centrality calculation function would be deactivated, requiring manual activation through the “compute node centrality” function in the node menu.

## 3 Results

### 3.1 Annual publications

**Figure 1:**
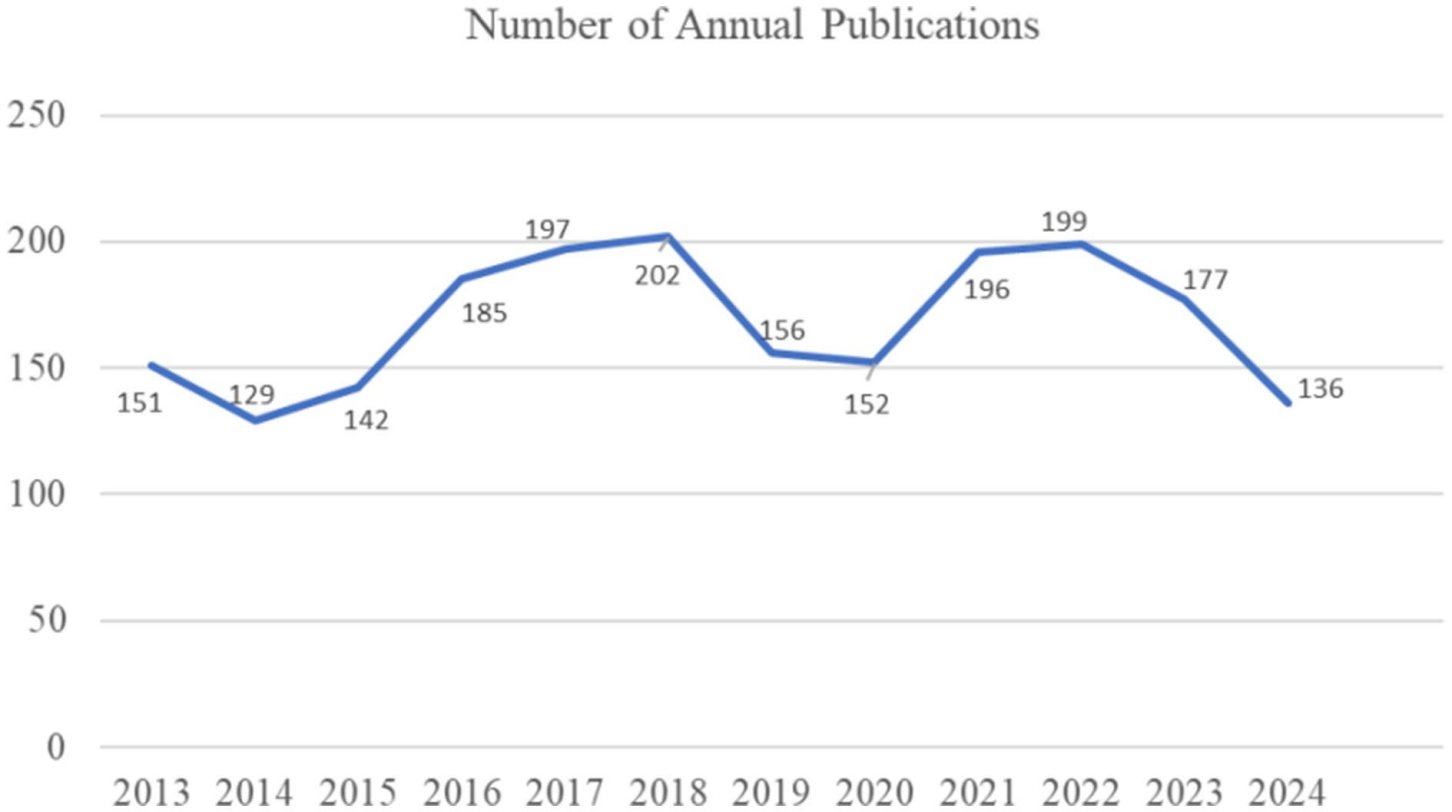
The number of annual publications on neuropathic pain after spinal cord injury research from 2013 to 2024.

A total of 2022 publications were retrieved. The number of annual publications is illustrated in Figure 1. Over the past 12 years, the number of publications has fluctuated but generally followed an upward trend; the highest number known is 202 in 2018. Since 2024 is not yet over, it is temporarily impossible to count its final number of publications. As depicted in Figure 1, the number of publications shows three declining stages and two rising stages. The decline in the number of publications occurred from 2013 to 2014, from 2019 to 2020, and from 2023 to 2024, the periods of rising publication numbers appeared from 2015 to 2018 and from 2021 to 2022. From 2015 to 2018, the number of publications increased from 142 to 202, accounting for 35.91% of the total publications over the 12 years. This indicates a growing interest among scholars in NP after SCI during this period.

### 3.2 Analysis of journals and cited journals

The number of journals that published the 2022 articles on NP after SCI was 543. Many journals are professional journals related to neuroscience and pain, while others are journals that cover rehabilitation, molecular and biological cell research, pharmacology, and professional diseases. The top 10 journals are listed by the number of publications in Table 1. The 10 journals published 446 articles, which account for 22.06% of the total records. These journals mostly belong to the neuroscience and pain journal category, which have made important contributions to the research progress of neuropathic pain. *Spinal Cord* ranked first in the frequency and the first impact factor (IF) ranking are *Pain* and *Neural Regeneration Research*. Two journals’ impact factor exceeded 5, whereas the average impact factor of the remaining journals was approximately 3.025. In addition, in accordance with the journal IF quartile of (13), we build a JCR journal quartile rankings. *Journal of Neurotrauma, Pain, Experimental Neurology, Neural Regeneration Research, European Journal of Pain,* and *Journal of Neuroscience* are categorized as Q1, which are highly regarded and very selective in what they publish, and the articles are worth studying. *Frontiers in Molecular Neuroscience* and *Journal of Pain Research* are categorized as Q2. *Spinal Cord* and *Journal of Spinal Cord Medicine* are categorized as Q3. For journals with categorized as low or low impact factor, the citation frequency is generally lower, and the quality of their articles may be difficult to assess. Therefore, it is important to make a careful judgment regarding the reference value of articles. For example, a retrospective study (14) published in the *Journal of Pain Research* by Xu et al. demonstrated that patients with neuropathic pain after SCI exhibit favorable outcomes in self-care, respiratory and sphincter management, and activity ability. This study provides a foundation for further exploration of the intensity and functional recovery of NP after SCI and provided confidence for clinical rehabilitation treatment.

**Table 1:** Top 10 most productive journals.

Rank	Journals	Frequency	Impact factor	JCR (2023)
1	Spinal Cord	80	2.1	Q3
2	Journal of Neurotrauma	70	3.9	Q1
3	Pain	51	5.9	Q1
4	Experimental Neurology	50	4.6	Q1
5	Journal of Spinal Cord Medicine	45	1.8	Q3
6	Neural Regeneration Research	32	5.9	Q1
7	European Journal of Pain	30	3.5	Q1
8	Frontiers in Molecular Neuroscience	30	3.5	Q2
9	Journal of Neuroscience	29	4.4	Q1
10	Journal of Pain Research	29	2.5	Q2

**Figure 2:**
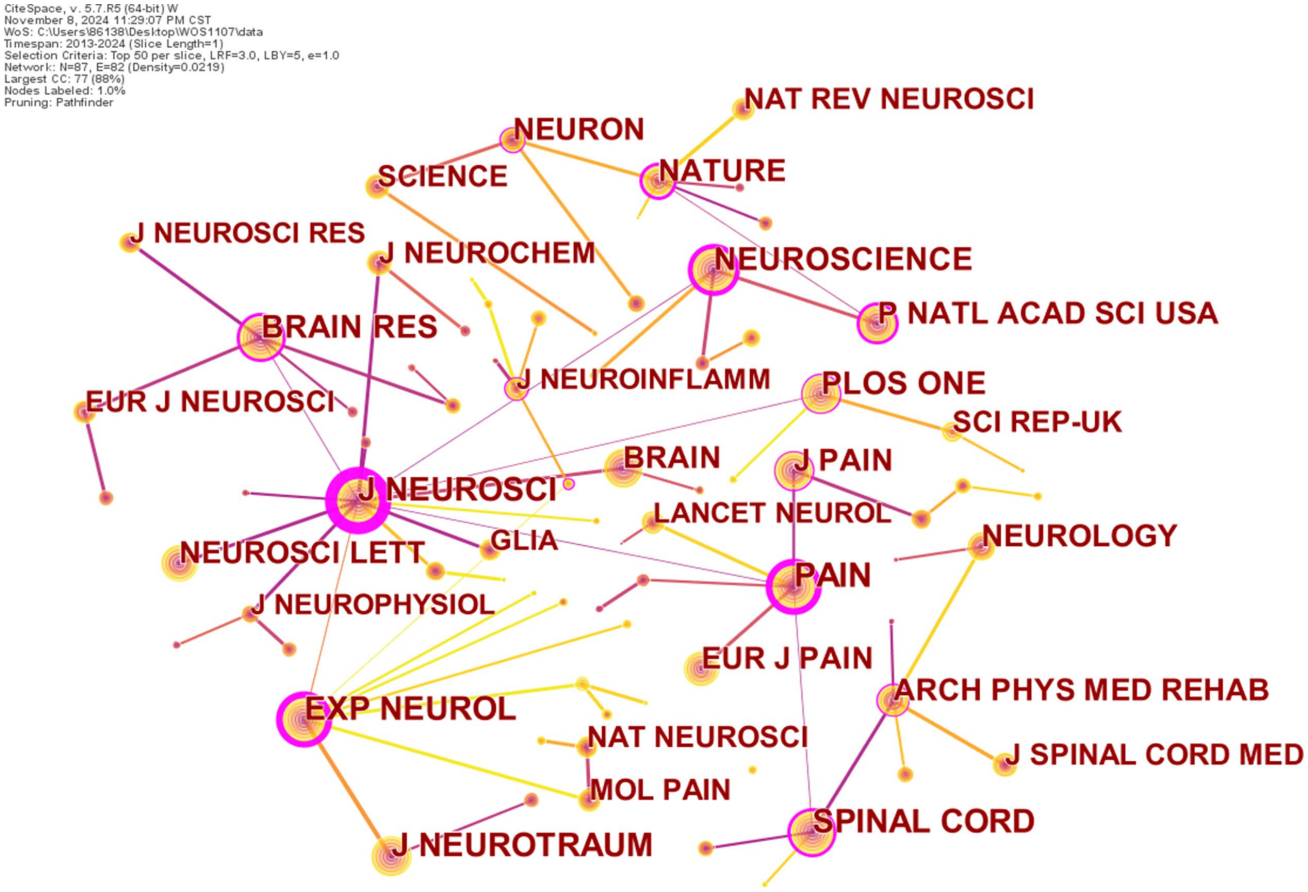
A cited journal map related to pain catastrophizing from 2013 to 2024. The nodes in the map represent the journal. The lines between the nodes represent cooperation relations.

We then generated a journal co-citation map to detect and evaluate influential journals that contribute to the development of NP after SCI research and serve as the knowledge base to some degree, as shown in Figure 2. CiteSpace configurations were set up as follows: Top *N* (*N* = 50) per year (2013–2024), LRF, link retaining factor = 3, LBY, look back year = 5, and e = 1. Sliced and merged networks were pruned by the Pathfinder algorithm, which resulted in 87 nodes and 82 links. The node sizes of cited journals and linkage thickness indicate citations and co-citations, respectively. From Figure 2, we can identify cited journals with high citations and/or with high betweenness centrality and draw out influential journals.

Co-citation analysis, one of the most important indicators, has been widely applied in bibliometrics. Co-cited journals were those cited together by other researchers. Through co-citation of journal analysis, we can obtain a distribution of key knowledge sources in a field. Table 2 presents the top 10 cited journals with the highest frequency and centrality of NP after SCI research. The most frequently cited journal was *Pain* (1510), followed by *Journal of Neuroscience* (1149) and *Experimental Neurology* (1024). In terms of centrality, the journals at top whose centrality exceeded 0.3 include the *Journal of Neuroscience* (1.31), *Pain* (0.63), *Experimental Neurology* (0.61), *Neuroscience* (0.47), *Proceedings of the National Academy of Sciences USA* (0.33), and *Nature* (0.31), which act as bridges linking other journals to a large extent. It is noteworthy that the top 10 sources of publications and most cited journals overlap to some extent, such as *Pain*, *Journal of Neuroscience*, *Experimental Neurology*, *Spinal Cord*, *Neuroscience*, and *Brain Research*, which can be deemed as reference journals for NP after SCI research.

**Table 2:** Top 10 journals with high citation frequency and high centrality.

Rank	Frequency	Centrality	Cited Journal	Frequency	Centrality	Cited journal
1	1,510	0.63	Pain	1,149	1.31	Journal of Neuroscience
2	1,149	1.31	Journal of Neuroscience	1,510	0.63	Pain
3	1,024	0.61	Experimental Neurology	1,024	0.61	Experimental Neurology
4	1,008	0.3	Spinal Cord	892	0.47	Neuroscience
5	994	0.04	Journal of Neurotrauma	743	0.33	P Natl Acad Sci USA
6	892	0.47	Neuroscience	623	0.31	Nature
7	892	0.12	PLoS One	1,008	0.3	Spinal Cord
8	877	0.24	Brain Research	877	0.24	Brain Research
9	799	0.04	Brain	670	0.2	Arch Phys Med Rehab
10	789	0.12	Journal of Pain	189	0.19	Front Cell Neurosci

We analyzed the co-citation relationship between *Pain* and *Journal of Neuroscience*, the top two journals in terms of centrality and citation frequency. The analysis of co-cited literature revealed that research trends in NP following SCI may focus on exercise therapy, neuroelectrophysiology, mechanoreceptors, glial cells, the spinal cord, thalamic function, and molecular mechanisms in the future.

### 3.3 Analysis of authors and cited authors

Concerning the number of publications, Armin Curt from the Spinal Cord Injury Center of the University Hospital of Balgris in Zurich, Switzerland, was the most prolific author. One of his articles identifying the metabolic NP signature after SCI by magnetic resonance spectroscopy provides new NP treatment targets (15). Michael G Fehlings, Wu Junfang, John L K Kramer, and Farinaz Nasirinezhad were also active in the field of pain catastrophizing research in Table 3.

**Table 3:** The top five authors.

Rank	Frequency	Centrality	Year	Author
1	23	0	2015	Armin Curt
2	14	0	2014	Michael G. Fehlings
3	14	0	2013	Junfang Wu
4	13	0	2015	John L. K. Kramer
5	12	0	2015	Farinaz Nasirinezhad

**Figure 3:**
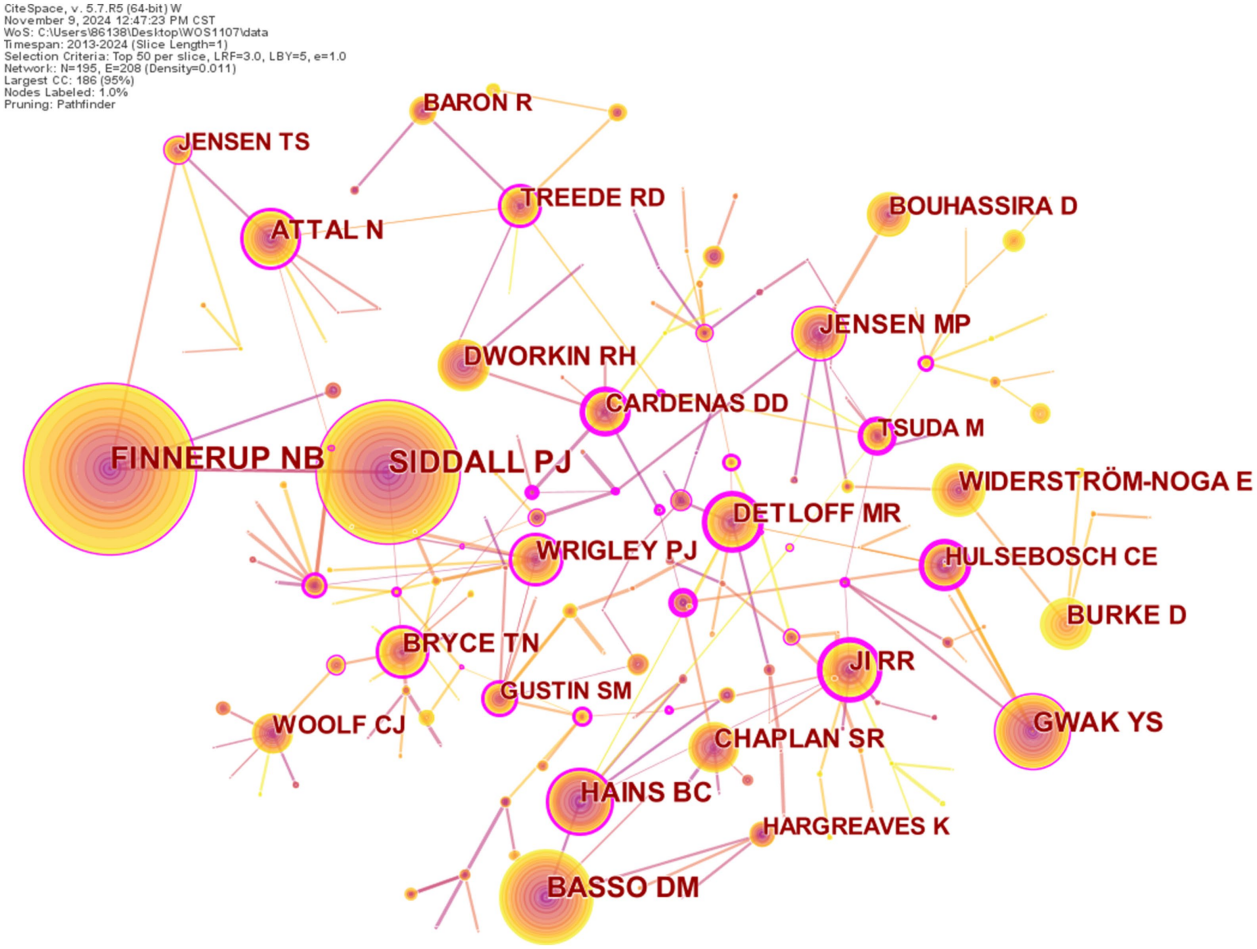
Author co-citation network.

**Figure 4:**
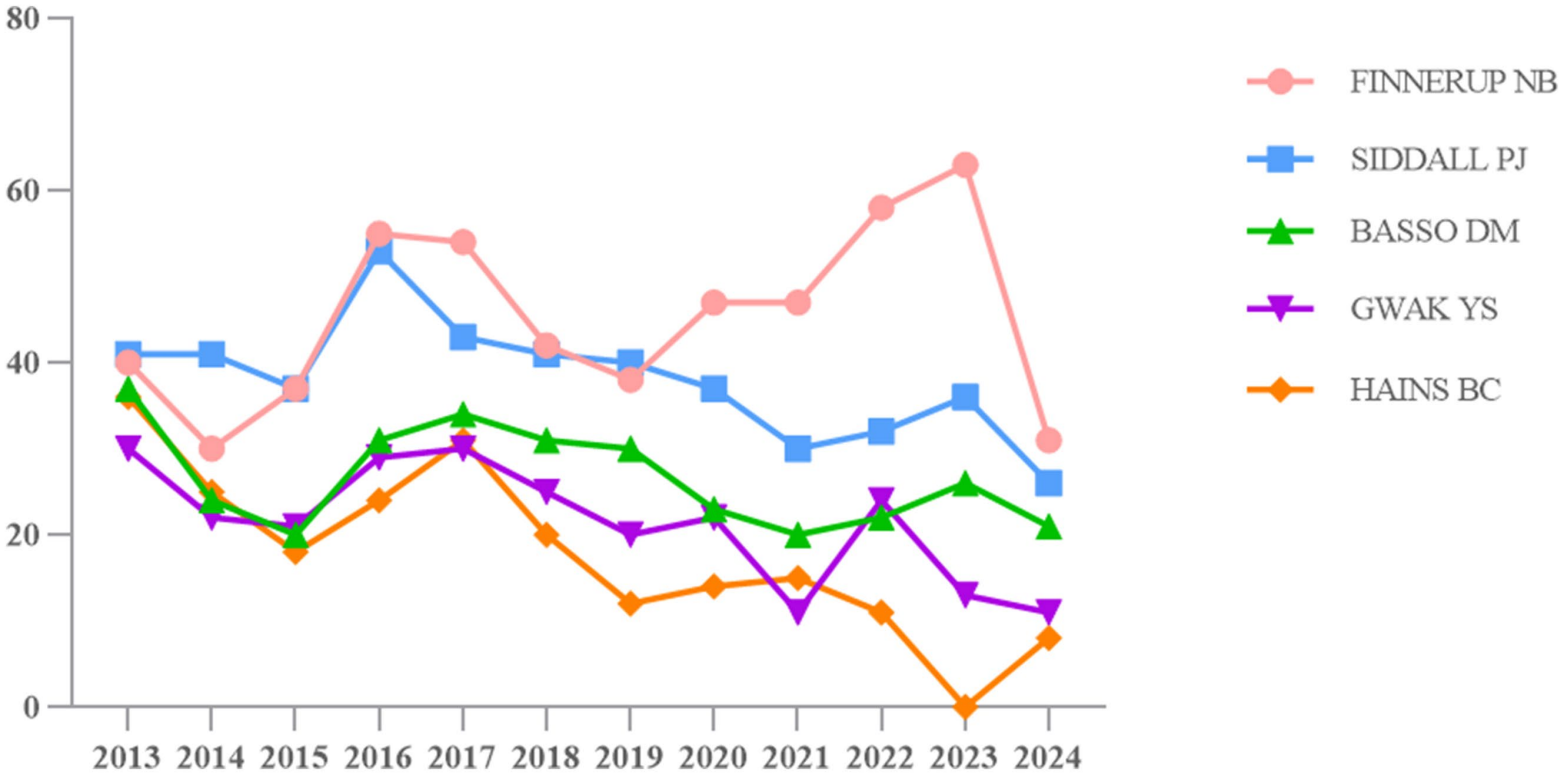
Citation distribution of the top five most cited authors.

Author co-citation network is generated to identify highly cited scholars whose publications are widely recognized by research communities in NP research after SCI. As shown in Figure 3, the map of the authors comprised 195 nodes and 208 links. When the size of the nodes is more larger the more times the author has, and the thickness of the lines between two node reflects the more times two authors are cited in the same article. The nodes with betweenness centrality no less than 0.1 are covered by a purple circle, and we can identify cited authors with high citations and/or with high betweenness centrality and draw out leading researchers. From Figure 3, the top five most highly cited authors are Nanna Brix Finnerup (542), Philip J. Siddall (457), D. Michele Basso (319), Young S Gwak (258), Bryan C. Hains (214). In terms of betweenness centrality, the top five are Megan Ryan Detloff (0.52), Makoto Tsuda (0.49), Diana D Cardenas (0.44), Claire E Hulsebosch (0.43), and John D Putzke (0.43). In addition, Table 4 gives a summary. Figure 4 shows the citation distribution of top five most cited authors. From Figure 4, we can know that on the whole, the citations of these five scholars fluctuated from 2013 to 2024, but it can be seen that the citation frequency of Nanna Brix Finnerup and Philip J. Siddall was significantly higher than that of the other three in the past 12 years.

**Table 4:** The top five cited authors with high frequency or betweenness centrality.

Rank	Frequency	Centrality	Cited author	Frequency	Centrality	Cited author
1	542	0.15	Finnerup NB	187	0.52	DETLOFF MR
2	457	0.15	Siddall PJ	112	0.49	TSUDA M
3	319	0.08	Basso DM	143	0.44	CARDENAS DD
4	258	0.18	Gwak YS	153	0.43	HULSEBOSCH CE
5	214	0.37	Hains BC	18	0.43	PUTZKE JD

Nanna Brix Finnerup works at Aarhus University and has been awarded the title of highly cited researcher in the field of neuroscience and behavior for four consecutive years (from 2020 to 2023). His research fields mainly include neurosciences and neurology, anesthesiology, rehabilitation, endocrinology and metabolism, and general and internal medicine. Philip J. Siddall works at Greenwich Hospital from Sydney in Australia, his research fields include neurosciences and neurology, rehabilitation, anesthesiology, general and internal medicine, health care sciences & services. Young S. Gwak works at the Department of Physiology, Korean Medical University, Daegu, Republic of Korea, whose expertise areas include food science and technology, biochemistry and molecular biology, and chemistry. D. Michele Basso works at the Ohio State University in USA and his research areas include neurosciences and neurology, rehabilitation, general and internal medicine, sport sciences, and orthopedics. Bryan C. Hains works at the University of Texas Medical Branch and his expertise areas include neurosciences and neurology, general and internal medicine, physiology, psychiatry, and surgery.

In terms of betweenness centrality, there are 23 scholars whose betweenness centrality is no lower than 0.2, indicating they are more influential than other scholars and exert a great impact on the development of NP after SCI research. For example, cited authors with the highest betweenness centrality Megan Ryan Detloff is a scholar in the field of neurosciences and neurology. Her research on NP after SCI has been extensively cited by other scholars to elucidate relevant pathological mechanisms and to explore new treatment approaches. For instance, Sliwinski et al. (16) referenced Detloff’s article “Acute Exercise Prevents the Development of NP and the Sprouting of Non-peptidergic (GDNF- and Artemin-responsive) C-fibers After Spinal Cord Injury.” Liu et al. (17) cited Detloff’s article, “Exercise-Induced Changes to the Macrophage Response in the Dorsal Root Ganglia Prevent Neuropathic Pain after Spinal Cord Injury.” Lee et al. (18) also referred to Detloff’s article, “Chronic at- and Below-level Pain After Moderate Unilateral Cervical Spinal Cord Contusion in Rats.”

In addition, we can also identify influential scholars from the point of citation bursts, that is, a scholar is cited much during a short period, which can be considered as major milestones in the development and of evolution NP search (19). The citations of several authors have been bursting to present, including Burke D. (with a burst strength of 33.65, from 2020), Ahuja CS (22.26, 2020), Li Y (21.44, 2021), Shiao R (18.92, 2022), and Zhang Y (18.57, 2020). The publications of these authors are worth studying because of their significant impact on NP research after SCI.

### 3.4 Analysis of cited references

**Figure 5:**
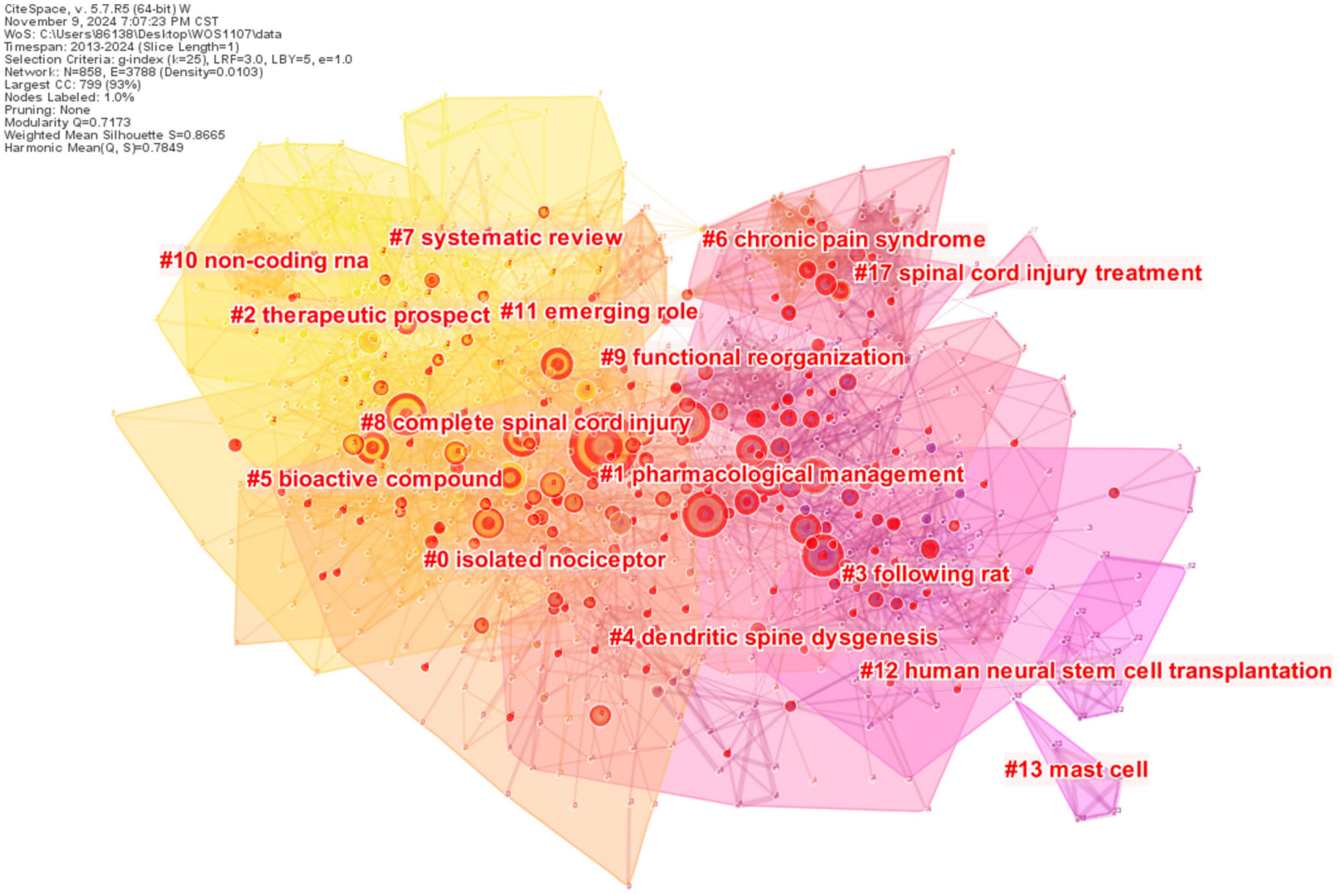
Cluster diagram of references.

When a group of documents is frequently cited in conjunction with other documents, this cluster may represent a certain research theme. Compared with other clusters, each cluster member is cited more frequently by a group of the same citing articles. In this section, based on 85,338 valid references cited in the 2022 records in our dataset, we applied the method of document co-citation analysis to visualize the landscape view of the NP field after SCI and analyze underlying knowledge base and research fronts. As shown in Figure 5, there are 15 clusters, including #0 isolated nociceptor, #1 pharmacological management, #2 therapeutic prospect, #3 following rat, #4 dendritic spine dysgenesis, #5 bioactive compound, #6 chronic pain syndrome, #7 systematic review, #8 complete spinal cord injury, #9 functional reorganization, #10 non-coding RNA, #11 emerging role, #12 human neural stem cell transplantation, #13 mast cell, #17 spinal cord injury treatment, which are major specialties of the NP after SCI field. Each cluster signifies distinct aspects of NP issues and topics. For these clusters, the color of the convex hull of each cluster indicates the mean year calculated on publication year of the cluster’s members. In addition, the brighter the color is, the closer the average year of one cluster is to the present. The quality of co-citation clusters is supposed to meet both criteria of modularity and weighted mean silhouette, which deserves to be thoroughly investigated. The modularity of the network is 0.7173, which is considered as a higher value, denoting that a well-structured network is developed and the specialties in NP fields after SCI are clearly defined. The weighted mean silhouette, as an indicator measuring the internal homogeneity of each cluster, is 0.8665, signifying the clustering is highly reliable and members of the corresponding cluster are more similar than other clusters’ members.

**Figure 6:**
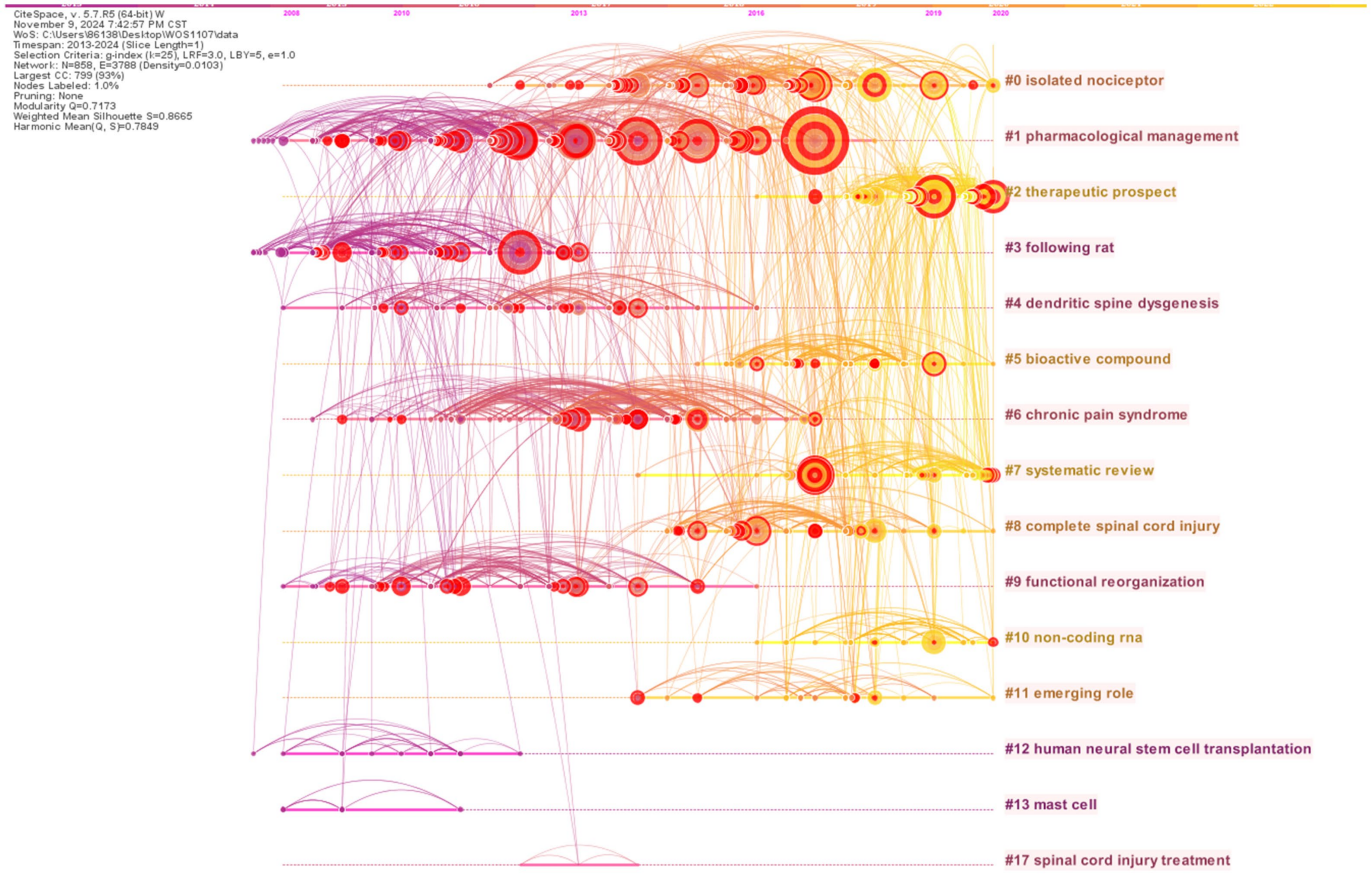
Timeline visualization of reference clustering.

As shown in Figure 6, all clusters are displayed on a timeline axis, which shows the research directions of NP after SCI. Each cluster’s members is shown in chronological order along the horizontal axis, whereas clusters are displayed vertically from top to down according to their sizes. From Figure 6, the duration of cluster # 1 has expired in 2018, but the cluster has a lot of literature worth studying.

Table 5 lists the details of these 15 clusters, and their silhouette values are greater than 0.7, which indicates that clusters are highly reliable and members have high internal consistency. Each of the three largest clusters has over 100 members. The largest cluster is #0 isolated nociceptor composed of 111 nodes, which accounted for 12.94% of the whole network. Then cluster #1 pharmacological management contains 110 nodes, accounting for 12.82% of the entire network, followed by cluster #2 therapeutic prospect composed of 107 nodes, accounting for 12.47% of the whole network. There are seven clusters whose durations exceed 5 years, including #0 isolated nociceptor, #1 pharmacological management, #4 dendritic spine dysgenesis, #6 chronic pain syndrome, #7 systematic review, #9 functional reorganization, and #11 emerging role. Among them, cluster #1 pharmacological management is the longest period, lasting 10 years. In terms of activeness, in Table 5, we can see that the latest time of the cluster is up to 2020, but this does not mean that researchers have lost interest in this field. Maybe they have explored new research directions in related fields (19).

**Table 5:** Detailed information about the 12 largest clusters.

Cluster ID	Size	%	Silhouette	Star	Stop	Mean (year)	Label (LLR)
0	111	12.94	0.783	2012	2020	2015	Isolated nociceptor
1	110	12.82	0.803	2008	2018	2012	Pharmacological management
2	107	12.47	0.796	2016	2020	2019	Therapeutic prospect
3	74	8.62	0.893	2008	2013	2010	Following rat
4	65	7.58	0.922	2008	2016	2012	Dendritic spine dysgenesis
5	61	7.12	0.871	2015	2020	2017	Bioactive compound
6	58	6.76	0.951	2009	2017	2013	Chronic pain syndrome
7	52	6.06	0.918	2014	2020	2018	Systematic review
8	51	5.94	0.882	2015	2020	2016	Complete spinal cord injury
9	41	4.78	0.956	2008	2016	2011	Functional reorganization
10	24	2.8	0.927	2016	2020	2018	Non-coding RNA
11	23	2.68	0.934	2014	2020	2017	Emerging role
12	13	1.52	0.994	2008	2012	2010	Human neural stem cell transplantation
13	6	0.7	0.999	2008	2011	2009	Mast cell
17	3	0.35	0.992	2012	2014	2013	Spinal cord injury treatment

Considering the size and activity of the cluster, we mainly focused on cluster #0 isolated nociceptor, cluster #2 therapeutic prospect, and cluster #7 systematic review.

Cluster #0, labeled as ‘Isolated Nociceptor,’ is the largest cluster, comprising 111 members across an eight-year period from 2012 to 2020. A study by Shiao et al. (20) has the most citations and the strongest burst strength within this cluster. They proposed that quantitative sensory testing could help distinguish between the mechanisms underlying low-grade NP and spasm, potentially offering new clinical options for identifying optimal treatments for NP after SCI. The most active citer to the cluster is Jonghoon Kang (21), whose article was cited in 23 articles within the cluster. This article reviews the current understanding of excessive neuronal excitability and maladaptive nociceptive transmission throughout the nervous system, which contributes to chronic central nervous system pain. A study by Li et al. (22) in 2020 showed that exercise training can ameliorate NP in rats with SCI. Therefore, we speculate that the research direction of this cluster in the field of NP after SCI is changing.

Cluster #2, labeled by therapeutic prospect, is the third-largest cluster, containing 110 nodes over a four-year period from 2016 to 2020. The treatment of NP after SCI remains a significant challenge, and the prospects for effective treatments continue to be a concern. Alizadeh et al. (23) review the pathophysiological progress of SCI, discussed the research results, and provided insights into future directions for NP research following SCI. Anjum et al. (24) pointed out numerous therapeutic strategies have been proposed to overcome neurodegenerative events and reduce secondary neuronal damage. However, due to the complexity of treatment, achieving satisfactory results remains difficult. Therefore, continued research is essential to identify more effective treatments for NP after SCI. In 2020, there was a noticeable shift toward focusing on the characteristics and evaluation of NP after SCI. For example, Pfyffer et al. (25) explored the correlation between tissue bridges and the development of NP through image analysis. Kim et al. (26) studied the prevalence and characteristics of NP in patients after SCI. The discussion of the characteristics and influencing factors of NP after SCI may lead to optimized management strategies and improved therapeutic outcomes.

Cluster #7, labeled “Systematic Review,” consists of 52 nodes. The most cited article in this cluster is published by Ahuja et al. (27), which discussed the key aspects of epidemiology, pathophysiology, and patient presentation in SCI. Additionally, the article outlines treatment strategies and future research directions. Colloca et al. (28) provide a detailed description of the presentation, causes, diagnosis, and treatment of NP, emphasizing the necessity of a multidisciplinary approach for the effective management of NP.

### 3.5 Analysis of keywords

**Figure 7:**
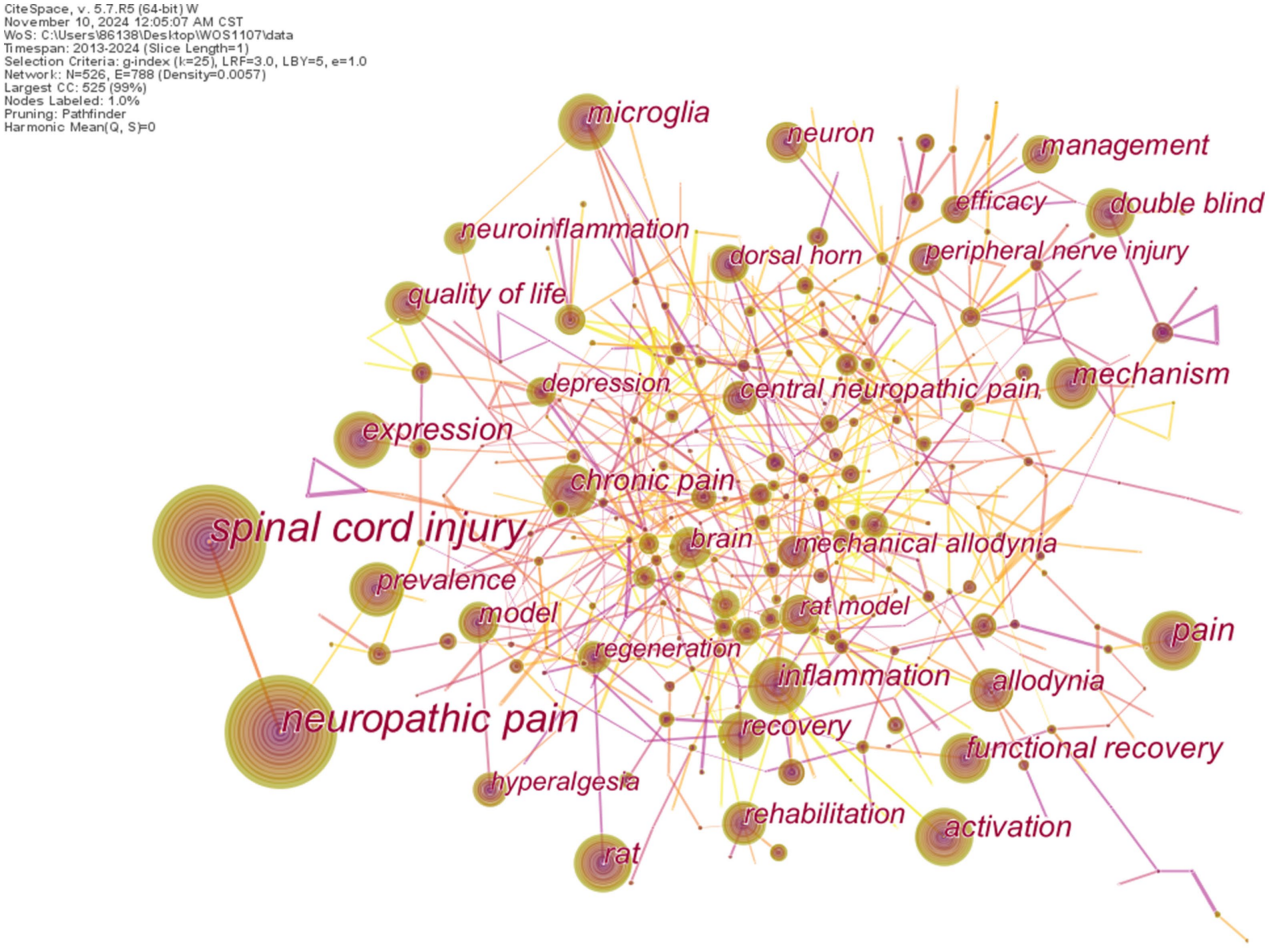
Keyword co-occurrence network.

Keywords are the concentration and refinement of the core content of the article, which contribute to efficient information retrieval and guides researchers to understand the core and essence of research (29). As shown in Figure 7, a node corresponds to a keyword and the size of the node represents the co-occurrence frequency of keywords. The link represents the time when two key terms appear in the same article. The brighter the color of the link, the closer the co-occurrence time is to the present (11, 30). We can evaluate the importance of a keyword by co-occurrence frequency and centrality. Relying on high-frequency and high-centrality keywords, the frontier content and research trends of the present field can be detected. Table 6 lists the top 10 keywords with high centrality or high frequency.

**Table 6:** Top 10 keywords with high count or centrality.

Rank	Frequency	Keywords	Centrality	Keywords
1	1,330	Spinal cord injury	0.25	Direct current stimulation
2	1,140	Neuropathic pain	0.18	Transcranial magnetic stimulation
3	223	Pain	0.15	Anxiety
4	186	Expression	0.14	Cortex
5	182	Activation	0.12	Apoptosis
6	179	Rat	0.12	Virtual reality
7	164	Inflammation	0.11	Motor cortex
8	162	Mechanism	0.11	Brain activity
9	151	Microglia	0.1	Efficacy
10	143	Functional recovery	0.1	Central nervous system

From the frequency point of view *spinal cord injury* with the frequency of 1,330 ranks first of all the keywords. The second highest count was *neuropathic pain* (1140), followed by *pain* (223). The keywords *expression* (186), *activation* (182), *inflammation* (164), *mechanism* (162), and *microglia* (151) represent the related mechanism of NP after SCI studied by scholars. *Rat* (129) represents the method of research. *Functional recovery* (143) represents the results of the study. In addition as shown in Figure 7 the most frequent keywords are *spinal cord injury* and *neuropathic pain*. These terms exhibit a strong co-occurrence relationship indicating a high likelihood of their simultaneous appearance in the literature.

At the level of centrality, the keywords with high centrality (centrality > = 0.1) are often regarded as inflection points in the keyword frequency knowledge graph and is of great significance in connecting other keywords or research topics, which to some extent represents significant themes or turning points in the research field. From Table 6, we can see that the centrality of the top 10 keywords is greater than 0.1, indicating that these keywords are worthy of our attention. Among them, *direct current stimulation* (0.25) has the highest centrality, followed by *transcranial magnetic stimulation* (0.18) and then *anxiety* (0.15).

**Figure 8:**
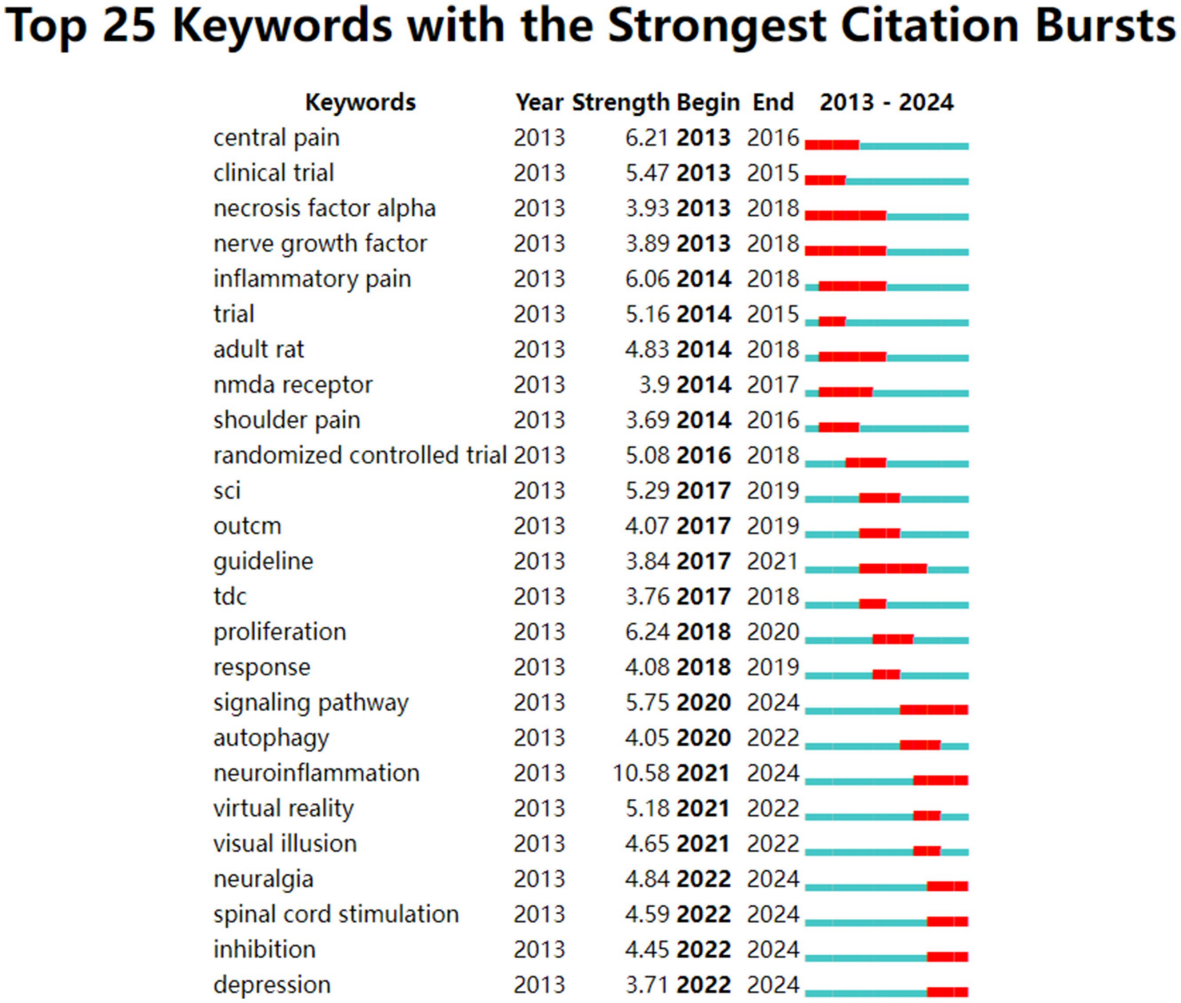
The top 25 keywords with the strongest citation bursts.

On the other hand, we also pay attention to the burst keywords, in order to identify the research hotspots in the field of NP after SCI. Figure 8 lists 25 keywords with strong bursts. In chronological order, the burst keywords in the NP after SCI field have been changing over the years from 2013 to 2024. The keywords with relatively long burst periods were *necrosis factor alpha* (2013–2018), *nerve growth factor* (2013–2018), with a duration of 6 years, which shows that these topics became research hotspots in the field of NP after SCI than other keywords during the same period. In addition, keywords such as *signaling pathway*, *neuroinflammation*, *neuralgia*, *spinal cord stimulation*, *inhibition*, and *depression* continue to be burst keywords until 2024. This reveals that researchers are currently focusing on the potential mechanisms, treatment methods, and mental health of NP after SCI.

From the point of view of burst strength, *neuroinflammation* (10.58) has the strongest burst, followed by *proliferation* (6.24), *central pain* (6.21), *inflammatory pain* (6.06), and *signaling pathway* (5.75), which are research hotspots in their corresponding periods.

## 4 Discussion

NP after SCI remains a challenging clinical problem, which has continued to attract the attention of researchers who are continuously seeking effective treatment methods. Existing treatment methods for NP after SCI include drug therapy (31), non-drug therapies [such as neuromodulation (32), physical therapy (33), acupuncture (34), psychotherapy (35, 36), and virtual reality (37), and surgical therapy (38)]. While drugs are commonly used, their effectiveness is limited, and they often cause significant side effects. Additionally, the development of new drugs has been slow. Non-drug treatments generally have fewer side effects, and new methods are being developed rapidly; however, most of these therapies are not supported by high-quality evidence. NP is a high-incidence symptom after SCI. Patients often experience significant anxiety and distress due to unbearable pain, leading to a serious reduction in their quality of life (3). However, there is currently no highly effective clinical method to help patients relieve this pain, and the existing research on its mechanism remains unclear, further increasing the difficulty of treatment. Over the years, scholars have been actively researching NP after SCI, which has garnered widespread academic attention.

However, the bibliometric literature that can help us better understand the research progress of NP after SCI is limited. The main purpose of this study is to visualize and conduct a systematic scientometric review of 2022 articles published from 2013 to 2024. In this study, the software CiteSpace 5.7.R5 was used to analyze the co-citation and co-occurrence of the literature retrieved from the WOSCC database. We concentrate on key nodes that exhibit high frequency, high betweenness centrality, or strong burst strength. By analyzing the publications, journals, authors, references, and keywords, we reveal the research hotspots and emerging trends in the field of NP after SCI during the same period. We hope that this article provides scholars with valuable insights into the overall research progress and serves as a useful reference for future studies.

Based on the analysis of journals and cited journals, authors and cited authors, and cited references network, the intellectual structure of the NP after SCI field is revealed.

The top 10 journals contributed to 22.06% (446) of the total number of publications on NP after SCI. The top five most productive journals include *Spinal Cord, Journal of Neurotrauma, Pain, Experimental Neurology*, and *Journal of Spinal Cord Medicine*. From the perspective of high centrality and high citation frequency, the influential journals in NP after SCI research include *Journal of Neuroscience, Pain, Experimental Neurology,* and *Neuroscience*. However, as shown in Table 1, among the top 10 journals, only two had an impact factor greater than 5, none had an impact factor over 10, but six were in the Q1 category. This indicates that publishing research on NP after SCI in more influential journals remains a challenge, but research in this field has attracted the attention of scholars.

The top five most highly cited authors are Nanna Brix Finnerup, Philip J. Siddall, D. Michele Basso, Young S. Gwak, and Bryan C Hains. With the exception of Young S. Gwak, the other four researchers’ areas of study are related to neuroscience and neurology, indicating that their research on NP after SCI has high reference value. Nanna Brix Finnerup (39) introduced the progress of SCI pain classification, the progress of understanding of potential mechanisms, and evidence-based SCI pain treatment in a review, providing a reference for exploring NP after SCI. In one of Philip J Siddall et al.’s (40) studies, the association between sensory pathways and neuralgia was tested under the injury plane through functional magnetic resonance imaging (fMIR) technology, which provides a technical means for improving the success rate of neuralgia after SCI and effectively utilizing residual function. A basic study by D. Michele Basso et al. (41) revealed the key role of microglia in the development of NP during RSD stress after SCI, suggesting that microglia could be targeted as a therapeutic approach to alleviate stress-related pain. In an early review by Bryan C. Hains et al. (42), the elucidation of molecular changes leading to the overexcitation of pain signal neurons may help identify molecular targets for treating NP and related neurological damage after SCI.

In the cluster diagram of references, we found that these clusters highlighted the topic of NP after SCI. From the timeline diagram of Figure 6, the latest duration of all clusters ends in 2020, which may indicate that scholars have adjusted the new direction of research on NP after SCI. These clusters include *isolated nociceptor, therapeutic prospect, systematic review, and non-coding RNA*. The emerging roles may provide valuable insights and guidance for future research directions. Areas such as *exercise therapy* (22, 43), *depression* (44), *neuroinflammation* (15), *quantitative sensory testing* (45), *glial cell* (46, 47), *neuroimaging* (48), *mechanism* (49, 50), and *pathophysiological* (21, 51) are likely to become new research trends. Due to the limitations in scientificity of experimental design, quality of evidence, sample size, subject homogeneity, and clinical feasibility in the corresponding studies, it is essential to enhance the reliability and applicability of the results in future research.

Keyword analysis reveals the conceptual structure of the NP after SCI domain. The keywords with high frequency in the top five are *spinal cord injury, neuropathic pain, pain, expression*, and *activation*. The top five keywords of high centrality are *direct current stimulation, transcranial magnetic stimulation, anxiety, cortex*, and *apoptosis*. The keywords with burst strength in the top five are *neuroinflammation, proliferation, central pain, inflammatory pain*, and *signaling pathway*, which are the research hotspots in the corresponding period. The keywords *signaling pathway*, *neuroinflammation*, *neuralgia*, *spinal cord stimulation*, *inhibition*, and *depression* continue to be new research hotspots, and signaling pathway lasted for 5 years. None of the other burst keywords continue for more than 5 years, which indicates that the research hotspots on NP after SCI may be constantly updated.

## 5 Conclusion and limitations

This study employs bibliometric methods to analyze key issues, research directions, and developmental trends in NP research after SCI from 2013 to 2024. A total of 2022 publications were included, with spinal cord ranking first in publication volume, and Armin Curt producing the highest number of articles. Keyword bursts indicate that topics such as signaling pathways, neuroinflammation, neuralgia, spinal cord stimulation, inhibition, and depression are likely to emerge as prominent research areas in the future.

There are several limitations in this study. Firstly, this study conducted data screening only from the WOSCC database. In the future, data collection and retrieval could be performed from additional databases to enable more rigorous screening and comprehensive data collection. Secondly, we only collected articles in English and excluded articles and reviews of other unexpected types of literature, which may introduce limitations to our results. Thirdly, we cannot rule out the possibility that our search strategies may not have been comprehensive, potentially leading to the omission of some relevant articles due to missing search terms. Although this study has its limitations, we are of the firm view that our review offers valuable insights and guidance for researchers and other readers in the field of NP after SCI.

In the future, we will address these issues. We may also consider combining bibliometric methods with traditional systematic reviews to analyze and integrate research on NP after SCI more comprehensively and accurately. In general, we hope that this article can provide a basis for the study of NP after SCI.

## Data Availability

The raw data supporting the conclusions of this article will be made available by the authors, without undue reservation.
